# Osteoglycin promotes meningioma development through downregulation of NF2 and activation of mTOR signaling

**DOI:** 10.1186/s12964-017-0189-7

**Published:** 2017-09-18

**Authors:** Yu Mei, Ziming Du, Changchen Hu, Noah F. Greenwald, Malak Abedalthagafi, Nathalie Y.R. Agar, Gavin P. Dunn, Wenya Linda Bi, Sandro Santagata, Ian F. Dunn

**Affiliations:** 1 Center for Skull Base and Pituitary Surgery, Department of Neurosurgery, Brigham and Women’s Hospital, Harvard Medical School, Boston, MA USA; 2 Department of Pathology, Brigham and Women’s Hospital, Harvard Medical School, Boston, MA USA; 3 Department of Neurosurgery, Shanxi Provincial People’s Hospital, Shanxi Medical University, Taiyuan, China; 4 Department of Cancer Biology, Dana-Farber Cancer Institute, Harvard Medical School, Boston, MA USA; 5 Saudi Human Genome Laboratory, King Fahad Medical City and King Abdulaziz City for Science and Technology, Riyadh, Saudi Arabia; 6 Department of Neurosurgery, Washington University School of Medicine, St. Louis, MO USA; 7 Center for Human Immunology and Immunotherapy Programs, Washington University School of Medicine, St. Louis, MO USA

**Keywords:** Osteoglycin, Autophagy, AKT inhibitor, Meningioma, Neurofibromatosis type 2, Mammalian target of rapamycin complex 1

## Abstract

**Background:**

Meningiomas are the most common primary intracranial tumors in adults. While a majority of meningiomas are slow growing neoplasms that may cured by surgical resection, a subset demonstrates more aggressive behavior and insidiously recurs despite surgery and radiation, without effective alternative treatment options. Elucidation of critical mitogenic pathways in meningioma oncogenesis may offer new therapeutic strategies. We performed an integrated genomic and molecular analysis to characterize the expression and function of osteoglycin (OGN) in meningiomas and explored possible therapeutic approaches for OGN-expressing meningiomas.

**Methods:**

*OGN* mRNA expression in human meningiomas was assessed by RNA microarray and RNAscope. The impact of OGN on cell proliferation, colony formation, and mitogenic signaling cascades was assessed in a human meningioma cell line (IOMM-Lee) with stable overexpression of OGN. Furthermore, the functional consequences of introducing an AKT inhibitor in OGN-overexpressing meningioma cells were assessed.

**Results:**

OGN mRNA expression was dramatically increased in meningiomas compared to a spectrum of other brain tumors and normal brain. OGN-overexpressing meningioma cells demonstrated an elevated rate of cell proliferation, cell cycle activation, and colony formation as compared with cells transfected with control vector. In addition, NF2 mRNA and protein expression were both attenuated in OGN-overexpressing cells. Conversely, mTOR pathway and AKT activation increased in OGN-overexpressing cells compared to control cells. Lastly, introduction of an AKT inhibitor reduced OGN expression in meningioma cells and resulted in increased cell death and autophagy, suggestive of a reciprocal relationship between OGN and AKT.

**Conclusion:**

We identify *OGN* as a novel oncogene in meningioma proliferation. AKT inhibition reduces OGN protein levels in meningioma cells, with a concomitant increase in cell death, which provides a promising treatment option for meningiomas with OGN overexpression.

**Electronic supplementary material:**

The online version of this article (10.1186/s12964-017-0189-7) contains supplementary material, which is available to authorized users.

## Background

Meningiomas represent approximately one-third of all primary brain tumors in adults and arise from the meninges surrounding the brain and spinal cord [1]. The World Health Organization (WHO) classifies meningiomas as grade I (benign), grade II (atypical), and grade III (anaplastic/malignant), with 15 histologic subtypes [2]. Most meningiomas are benign (90% grade I) and slow growing, with effective control following surgical resection if treatment is indicated. However, grade II-III meningiomas and those located at the skull base offer a management challenge due to their predilection for recurrence and premature morbidity and mortality from disease, despite surgery and radiation. The lack of effective pharmacotherapeutic options motivates further definition of meningioma biology to provide alternative strategies for treatment.

Meningiomas represent one of the first tumors to be associated with a genomic driver with the discovery that Neurofibromatosis 2 (NF2), an inherited genetic disorder characterized by the development of schwannomas and meningiomas, arose in the setting of mutations of the *NF2* gene [3]. Recent next-generation genomic analysis of meningiomas revealed additional recurrent mutations in v-akt murine thymoma viral oncogene homolog 1/3 (*AKT1/3*), phosphoinositide-3-kinase catalytic alpha polypeptide (*PIK3CA*)*,* smoothened (*SMO*), homolog of suppressor of fused (*SUFU*)*,* TNF receptor-associated factor 7 (*TRAF7*), krupplelike factor 4 (*KLF4*), SWI/SNF related, matrix associated, actin dependent regulator of chromatin, subfamily b, member 1 (*SMARCB1*), RNA polymerase II subunit A (*POLR2A*), telomerase reverse transcriptase (*TERT*) promoter, and BRCA1 associated protein 1 (*BAP1*) [4–10]. These, as well as loss of Merlin, the protein product of *NF2*, are additionally associated with downstream activation of mitogenic pathways such as the mTOR and Hedgehog cascades, to produce uncontrolled neoplastic growth [11]. However, the precise mechanisms by which meningioma oncogenesis occurs remains incompletely understood.

Osteoglycin (OGN), located on 9q22.31, plays critical roles in both physiological condition, such as the formation of bone [12] and normal vasculature [13, 14], as well as pathological processes including vascular differentiation and remodeling [14]. Cytokines associated with vascular injury, such as basic fibroblast growth factor, transforming growth factor-beta, platelet-derived growth factor and angiotensin II, downregulate *OGN* gene expression [14]. Furthermore, OGN is a major regulator of ventricular hypertrophy [15]. However, whether OGN is involved in meningioma development is currently unknown.

We performed an integrated genomic and molecular analysis to define the expression of *OGN* in meningiomas, illustrate how OGN may contribute to meningioma cell growth through interaction with other drivers of meningioma formation such as NF2, AKT, and mTOR, and explore possible therapeutic approaches for OGN-expressing meningiomas.

## Methods

### Expression profiling of *OGN* in human meningiomas

The expression profiling data were normalized from publicly available datasets cataloged in GEO (Gene Expression Omnibus) that had been acquired on Affymetrix Human Genome U133 Plus 2.0 Array Platform [HG-U133_Plus_2], as previously described [16]. The following datasets were used: GSE16155 (ependymoma), GSE16581 (meningioma), GSE34824 (pediatric glioblastoma), GSE36245 (adult glioblastoma), GSE33331 (adult astrocytoma), GSE35493 (atypical, teratoid, rhabdoid tumors/ATRT; medulloblastoma) GSE19404 (primitive neuroectodermal tumor), GSE34771 (CNS lymphoma), GSE5675 (pilocytic astrocytoma).

### RNA scope in situ hybridization

Formalin-fixed, paraffin-embedded human meningioma specimens were collected from the Department of Pathology, Brigham and Women’s Hospital, with corresponding clinical records and pathology reports. Hematoxylin and eosin stained sections corresponding to each tumor were reviewed by two neuropathologists (SS, MA) for selection of specimens with greater than 70% estimated tumor purity. Two hundred seven meningiomas (with triplicate cores, spanning 621 samples total) were compiled in tissue microarray (TMA) format for subsequent analysis. The study was approved by the Institutional Review Boards of Brigham and Women’s Hospital and Dana Farber Cancer Institute, Harvard Medical School. In situ detection of OGN transcripts in meningioma TMA was performed using RNAscope® assay with Probe-Hs-OGN (Cat# 498831, Advanced Cell Diagnostics, Newark, USA) and RNAscope® 2.0 HD Reagent Kit (Cat# 310035, Advanced Cell Diagnostics) following manufacturer protocols. All slides were digitally scanned using Carl Zeiss Microimaging (Jena, Germany). RNAscope staining was quantified by NIH ImageJ software (Bethesda, USA). Single DAB stained images were obtained using color deconvolution as previously described [17]. After adjustment of the color threshold, the intensity of the DAB-positive staining was measured. Optical density-log (max intensity/mean intensity) was used for statistical analysis.

### Human meningioma cell line culture

The human meningioma cell line IOMM-Lee (derived from a grade III meningioma) [18, 19], courtesy of Dr. Randy Jensen (University of Utah), was cultured in growth media composed of RPMI 1640 Medium, 10% fetal bovine serum, 2 mM L-glutamine, 100 IU/mL of penicillin, and 100 μg/mL of streptomycin (Life Technology, Grand Island, USA). Cultured cells were maintained at 37° in a 5% CO_2_ atmosphere.

### OGN expressing stable cell line generation

IOMM-Lee cells were plated in 12-well plates at a density of 200,000 cells/well, and transfected with pCMV/Control Vector (C-terminal Fc-Myc-tagged) or pCMV/OGN Vector (Myc-tagged) (Sino Biological, Beijing, China). At 48 h after transfection, cells were trypsinized and plated in 96-well plates for selection. Monoclonal populations with stable expression of control vector (Control-IOMM) or OGN (OGN-IOMM) were selected with addition of hygromycin B (0.2 g/ml). Gene stability was verified for at least ten passages by analysis of OGN mRNA and protein expression.

### Cell proliferation assay

Meningioma cells with and without OGN expression were assessed for proliferation using the WST-1 assay (Roche, Indianapolis, USA), which is a nonradioactive method to quantify cell proliferation and survival. Briefly, Control-IOMM and OGN-IOMM cells were seeded in 96-well plates at a density of 10,000 cells/well and cultured for 72 h. Cells were then incubated with WST-1 for 2 h. Absorbance, as a measure of cell proliferation, was measured on an Epoch Microplate Spectrophotometer (BioTek, Winooski, USA).

### Soft agar growth assay

Control-IOMM and OGN-IOMM cells (250,000/well) were mixed with 0.4% agarose in growth medium, plated on top of a solidified layer of 0.5% agarose in a 6-well plate, and fed every 3 d with growth medium. After 18 days, the colonies were stained with 0.01% Crystal Violet (EMD, Billerica, USA) and imaged by Zeiss Axiovert 40 CFL Microimaging (Jena, Germany). Colony size and number were quantified using ImageJ (NIH, Bethesda, USA). Average colony sizes (total colony size/total colony number) were used for statistical analysis.

### Inhibitor assays

The impact of the AKT inhibitor AKTVIII and mTOR inhibitor rapamycin (Cayman Chemical, Ann Arbor, USA) on meningioma cells were assessed, with and without OGN overexpression. Briefly, OGN-IOMM cells were plated at a density of 400,000/well in 12-well plates and treated with rapamycin (10 μM), cells were harvested at 6 h after treatment and analyzed by Western blot for mTOR activation and OGN expression.

OGN-IOMM cells were seeded in 96-well plates at a density of 10,000 cells/well and treated with AKTVIII before incubation with WST-1 for 2 h for assessment of cytotoxic response. Serial dilutions of each drug were tested to determine an optimal concentration for synthetic lethality assessment at 24-72 h. Control-IOMM and OGN-IOMM cells were treated with AKT VIII to compare the response to inhibitor induced cell cytotoxicity. Absorbance was determined by Epoch Microplate Spectrophotometer (BioTek).

Furthermore, the influence of AKTVIII on signaling pathways was assessed in OGN-expressing IOMM-Lee meningioma cells. OGN-IOMM cells were plated at a density of 200,000/well in 12-well plates and treated with AKT VIII for 48 h, cells were harvested or fixed for protein expression by Western blot or immunohistochemistry.

### RNA knockdown

OGN-IOMM cells were plated at a density of 400,000/well in 12-well plates and transfected with control siRNA (Cat#12935-300, Thermal Fisher Scientific, Grand Island, USA), NF2 siRNA (cat#HSS143098, Thermal Fisher Scientific), or OGN siRNA (Cat#HSS107424, Thermal Fisher Scientific) as described by manufacturer protocol. Transfected cells were cultured for 24 or 48 h. Cells were then washed with PBS and harvested for mRNA or protein analysis.

### Quantitative real-time PCR

Quantitative real-time PCR (qPCR) was performed to assess expression of mRNA following specific culture conditions. Total RNA was extracted with the Aurum™ Total RNA Mini Kit (Bio-Rad, Hercules, USA) and cDNA was synthesized using the high capacity RNA-to-cDNA™ Kit (Life Technologies, Grand Island, USA) according to manufacturer instructions. qPCR was performed using gene-specific FAM-NFQ-conjugated TaqMan primers for human *OGN* and *NF2* (Thermal Fisher Scientific). Level of mRNA expression was normalized to β-actin. Expression was analyzed using the comparative cycle threshold (ΔΔCT) with Applied Biosystems 7300 Real Time PCR Software (Life Technology).

### Western blotting

Human meningioma samples were obtained from the Brigham and Women’s Hospital Department of Pathology, under institutional IRB approval. Protein from meningioma samples was extracted using RIPA buffer (Cell Signaling, Danvers, USA). Cultured meningioma cells with or without treatment were lysed using cell lysis buffer (Cell Signaling, Danvers, USA). 20 μg of protein from each sample was resolved by 4–20% SDS-PAGE (Bio-Rad), before transfer to a PVDF membrane for overnight incubation with primary antibodies at 4 °C. Primary antibodies were diluted at 1:1000 for OGN (Abcam, Cambridge, USA), cyclinD1, cyclinA2, cyclinB1, GAPDH, p-AKT(Ser473), p-mTOR (Ser2448), p-4E-BP1(Ser65), p-eIF4B (Ser422), p-eEF2K (Ser366), p-ULK(Ser757), NF2, LC3IIB (Cell Signaling, Danvers, USA). Membranes were then incubated with horseradish peroxidase-conjugated goat anti-rabbit or goat anti-mouse IgG (Cell Signaling, Danvers, USA) at 1:2000. Blots were visualized by an ECL system (GE Healthcare, Buckinghamshire, UK) and protein quantified by densitometry analyses using ImageJ.

### Immunohistochemistry

Protein expression of OGN was corroborated with immunohistochemistry (IHC). Control-IOMM and OGN-IOMM cells were cultured for 48 h, and then were fixed and washed with PBS. Cells were incubated with primary antibody specific to OGN (Abcam, Cambridge, USA), NF2 or anti-Rabbit IgG (Cell signaling, Danvers, USA), OGN-IOMM cells treated with AKT VIII for 48 h were fixed and incubated with anti-LC3BII, followed by horseradish peroxidase-conjugated secondary antibody (Vector lab, Burlingame, USA). Immunopositivity was visualized using DAB system (Vector Lab, Burlingame, USA). All slides were digitally scanned using Zeiss Microimaging (Jena, Germany).

### OGN correlation analysis

DNA sequencing data [20] of 35 meningiomas with OGN RNAscope data were reviewed. These tumors were grouped as OGN low (lower than average) and OGN high (higher than average) according to their OGN RNA level. The incidence of *NF2* or chromosomal 22 loss between the subgroups was compared. In addition, OGN protein level in meningiomas with intact NF2 or NF2 loss was assessed by Western blotting.

### Statistical analysis

All data obtained in vitro represent three independent experiments. Results are expressed as mean ± standard error. Statistical analysis was performed using Prism (GraphPad, La Jolla, USA), and comparisons were made with Student’s t-test, correlation, or ANOVA. A *p* value < 0.05 was considered significant.

## Results

### OGN gene expression is dramatically increased in human meningiomas

**Fig. 1:**
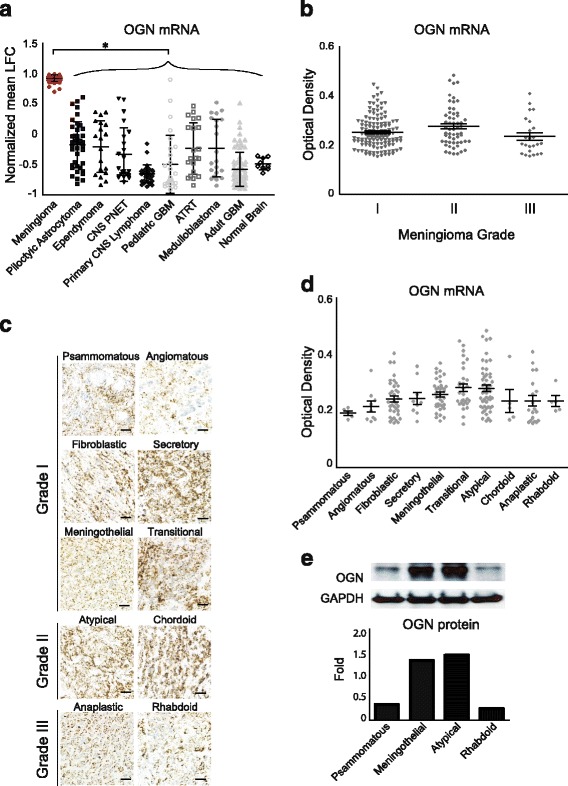
OGN gene expression is dramatically increased in human meningiomas. **a** *OGN* expression is significantly higher in human meningiomas compared to 8 other CNS tumors and normal brain, as detected by microarray, *p < 0.0001. **b** RNAscope staining reveals *OGN* mRNA expression across all grades of human meningioma. **c** RNAscope staining of OGN mRNA across multiple histologic subtypes and grades of human meningioma, scale bar 100 μm, with **d** optical density quantification. **e** Western blotting and densitometry analysis of OGN protein expression in multiple meningioma subtypes. CNS, central nervous system; LFC, log fold change; GBM, glioblastoma; OGN, osteoglycin, PNET, primitive neuroectodermal tumor; ATRT, atypical teratoid rhaboid tumor

To determine the expression level of OGN in meningiomas, microarray data for 9 brain tumors and normal human brain were analyzed. *As compared to negative control* (Additional file 1: Figure S1)*, OGN* mRNA expression was dramatically increased in meningiomas (68 samples) compared to 8 other CNS tumors and normal brain tissue (*p* < 0.0001, Fig. 1a).

To confirm this finding, RNAscope was performed to quantify *OGN* mRNA level in meningiomas across all grades (126 grade I, 57 grade II, 24 grade III, Fig. 1b, *p* = 0.0338) and pathology subtypes (Fig. 1c). Transitional and atypical meningiomas demonstrated the highest levels of *OGN* mRNA while psammomatous meningiomas demonstrated the lowest (Fig. 1d, *p* = 0.0041). In meningiomas with known treatment status, *OGN* mRNA expression levels in primary (74 samples) and recurrent (24 samples) tumors were comparable (data not shown). Consistent with the RNA level, OGN protein expression was higher in meningothelial and atypical subtypes, but lower in psammomatous and rhabdoid meningiomas as determined by Western blotting (Fig. 1e).

### OGN overexpression promotes cell proliferation and tumor cell colony formation

**Fig. 2:**
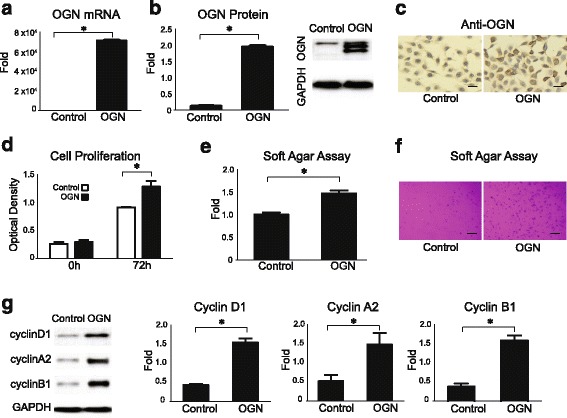
OGN overexpression promotes cell proliferation and tumor cell colony formation. **a** *OGN* mRNA is expressed in IOMM-Lee meningioma cells stably transfected with *OGN* vector as compared to cells transfected with control vector. **b** Western blot and densitometry analysis reveals significant expression of OGN protein in meningioma cells stably transfected with *OGN* vector. **c** Immunohistochemistry profile of OGN protein expression in control and OGN-expressing cells, scale bar 50 μm. **d** OGN-expressing cells demonstrate significantly increased proliferation at 72 h after plating, compared to control cells, as evaluated by WST assay. **e**, **f** After 18 days of incubation in soft agar, OGN-expressing cells demonstrate significantly increased colony formation, scale bar 100 μm. **g** Western blotting and densitometry analysis reveals higher expression of cell cycle markers, cyclin D1, cyclin A2, and cyclin B1 in OGN cells compared with control cells. *p < 0.05

To determine the mitogenic effects of OGN on meningioma cell proliferation, we stably transfected *OGN* constructs into IOMM-Lee, a malignant human meningioma cell line with minimal levels of endogenous OGN, with confirmation of mRNA expression by qPCR (Fig. 2a) and protein expression by Western blotting and IHC (Fig. 2b-c).

Functional assessment of OGN overexpression in vitro revealed a higher proliferation rate than control cells at 72 h (Fig. 2d) as well as more active tumor cell colony formation in soft agar (Fig. 2e-f). Corresponding to this increased proliferative behavior, higher levels of protein expression were observed for the cell cycle regulators cyclin D1, A2, and B1 in OGN-overexpressing cells compared to control cells (Fig. 2g).

### Knockdown of OGN reduces cell proliferation

**Fig. 3:**
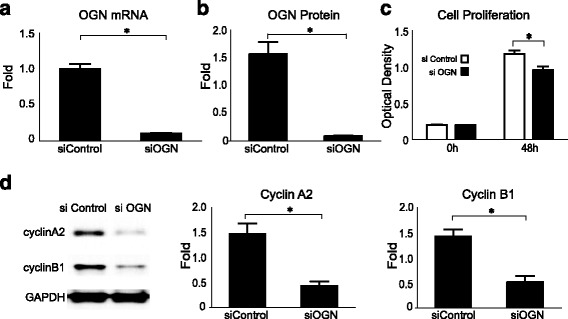
Knockdown of OGN reduces cell proliferation. OGN **a** mRNA expression at 24 h and **b** protein expression at 48 h are successfully reduced in OGN cells transfected with OGN siRNA (siOGN) compared with control siRNA (siControl) on qPCR and Western blot analysis, respectively. **c** Knockdown of OGN reduced cell proliferation as compared to control siRNA transfected cells. **d** Western blot and densitometry analysis reveals decreased expression of the cell cycle markers cyclin A2 and cyclin B1 at 48 h in cells with knockdown of OGN. *p < 0.05

To confirm the mitogenic effects of OGN on cell proliferation, OGN was knocked down in stably transfected cells using siRNA, with significant reduction in mRNA (Fig. 3a) at 24 h and protein expression (Fig. 3b) at 48 h after transfection. Meningioma cells with knockdown of OGN also demonstrated significantly reduced cell proliferation (Fig. 3c) and expression of the cell cycle markers cyclin A2 and cyclin B1 (Fig. 3d), compared to cells with stable expression of OGN.

### OGN downregulates NF2

**Fig. 4:**
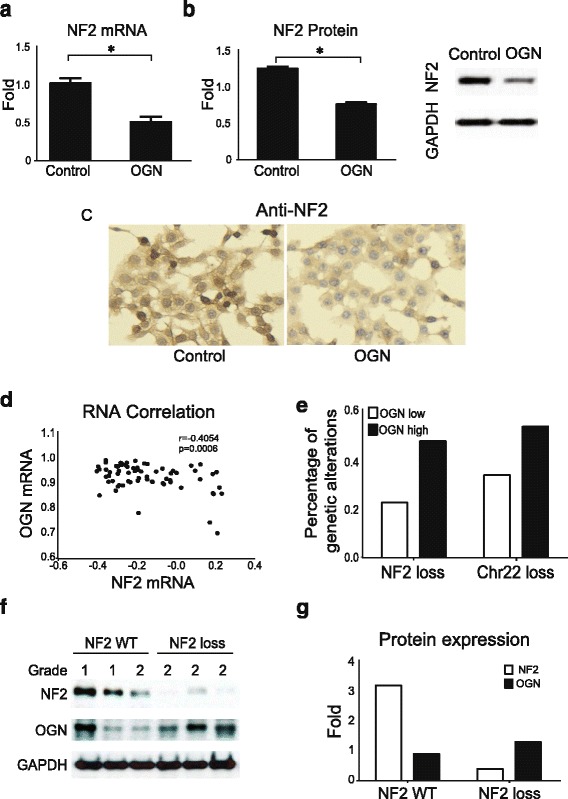
OGN downregulates NF2. NF2 **a** mRNA and **b** protein expression are reduced in OGN-expressing cells, compared to control meningioma cells on qPCR and Western blot analysis, respectively. **c** Control cells demonstrate more NF2 immunoreactivity compared to OGN cells. *p < 0.05, scale bar 50 μm **d** Correlation analysis of human meningioma RNA microarray data displays significantly negative correlation of NF2 mRNA to OGN mRNA. **e** Analysis of human meningiomas reveals increased frequency of *NF2* or chr22 loss in samples with higher *OGN* mRNA expression. **f** Western blotting analysis of OGN and NF2 protein expression in human meningiomas with intact (WT, wildtype) *NF2* or *NF2* loss in WHO grade 1 and 2 meningiomas. **g** Densitometry quantification displays NF2 loss tumors express low NF2 but higher OGN protein

Loss of function of the tumor suppressor NF2 plays a driver role in the meningioma initiation and development [21]. We performed whole-exome sequencing of the IOMM-Lee cell line and detected no mutations in *NF2* or loss of chromosome 22 [22]. To determine whether the effects of OGN in IOMM cells are mediated by NF2, NF2 expression at the transcription and translational level was evaluated by qPCR and Western blotting following transfection of *OGN*. *NF2* mRNA expression was attenuated in OGN-expressing meningioma cells (Fig. 4a), with corresponding subsequent decrease in NF2 protein level (Fig. 4b). Consistent with these findings, positive immunostaining for Merlin, the protein product of *NF2*, was observed in both groups, with attenuated expression in OGN-expressing cells (Fig. 4c).

To confirm the in vitro findings, correlation analysis of human meningioma RNA microarray data demonstrated a significant negative correlation between NF2 mRNA and OGN mRNA (Fig. 4d). We further analyzed 35 meningiomas with the whole exome or whole genome sequencing data [20], the frequency analysis of genetic alteration reveals increased *NF2* or chr22 loss in human meningiomas with higher OGN mRNA expression (Fig. 4e). To confirm the inverse relationship between OGN and NF2, human meningiomas with intact *NF2* or *NF2* loss were assessed for OGN protein expression, revealing that meningiomas with *NF2* loss express higher levels of OGN (Fig. 4f-g).

### OGN activates AKT and mTOR signaling

**Fig. 5:**
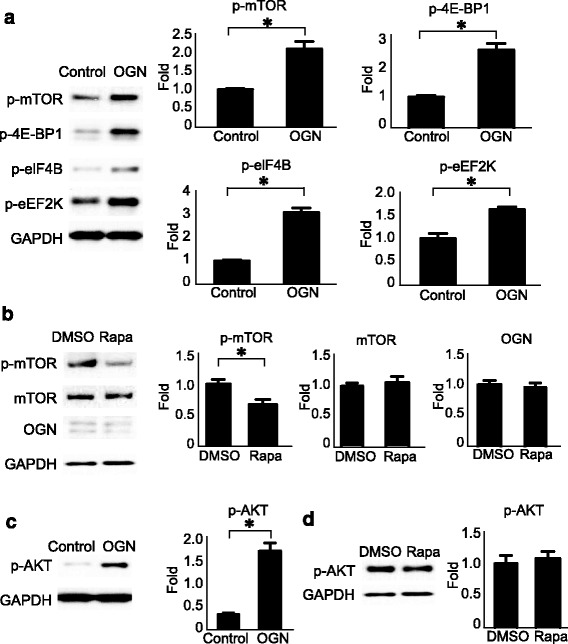
OGN activates mTOR signaling and AKT. **a** OGN-expressing cells demonstrate increased activation of mTOR and its downstream signaling effectors at 48 h, including p-mTOR (Ser2448), p-4E-BP1(Ser65), p-eIF4B (Ser422), and p-eEF2K (Ser366), compared to controls on Western blot analysis with densitometry quantification. **b** Treatment with the mTOR inhibitor rapamycin (Rapa) suppresses mTOR activation without affecting OGN expression in OGN cells, as compared to DMSO control at 6 h. **c** OGN-expressing cells also activate AKT (p-AKT Ser473) as early as 6 h after plating, on Western blot analysis with densitometry quantification. **d** Addition of rapamycin (Rapa) does not affect AKT activation at 6 h in OGN cells. *p < 0.05

Several biological pathways in meningioma oncogenesis, including loss of *NF2* and mutation of *AKT1*, are postulated to exhibit their oncogenic effects through activation of the mTOR mitogenic pathway, leading to uncontrolled neoplastic growth [4, 11]. To determine the effects of OGN on the mTOR complex cascade, we analyzed the activation of proteins involved in mTOR signaling. Western blotting analysis indicated that the phosphorylation of mTOR and its downstream signals, eukaryotic translation initiation factor 4E-binding protein 1 (4E-BP1), eukaryotic translation initiation factor 4B (eIF4B), and eukaryotic elongation factor 2 kinase (eEF2K), were all elevated in OGN-overexpressing meningioma lines (Fig. 5a). Quantification of the band densitometry confirmed significant increase in the expression of phospho-mTOR^Ser2448^, phospho-4E-BP1^Ser65^, phospho-eIF4B^Ser422^, and phospho-eEF2K^Ser366^ in OGN-IOMM cells compared with their expression in control cell lines. Conversely, introduction of the mTOR inhibitor rapamycin (10 μM) effectively reduced mTOR activation but did not alter OGN expression in OGN-IOMM cells (Fig. 5b), suggesting that OGN acts upstream of mTOR signaling.

Furthermore, stable expression of OGN in meningioma cells produced a significant increase in phospho-AKT (Fig. 5c). This activation of AKT by OGN is not affected by mTOR pathway inhibition with rapamycin (Fig. 5d).

### AKT inhibitor reduces mTOR activation and OGN expression in promoting cell death

**Fig. 6:**
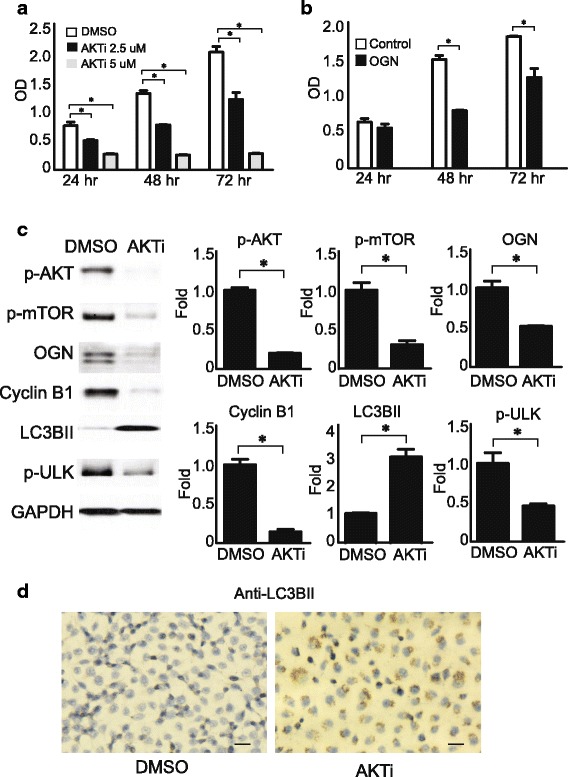
AKT inhibitor reduces OGN expression, mTOR activation, and promotes cell death. **a** OGN cells demonstrate decreased survival upon addition of AKT inhibitor (AKTi) in a time and dose dependent manner. **b** OGN-expressing meningioma cells demonstrate decreased survival compared to control cells upon addition of 2.5uM AKTi at 48 h and 72 h. **c** 2.5uM AKTi significantly reduces AKT and mTOR activation, OGN expression, cell cycle marker cyclin B1 expression, and the autophagy inhibitor ULK, while increasing expression of the autophagy marker LC3IIB in OGN cells at 48 h, compared to DMSO treatment. **d** Decreased cell survival with increased immunoreactivity for the autophagy marker LC3BII in OGN cells treated with 2.5uM AKTi compared to DMSO at 48 h. *p < 0.05, scale bar 50 μm

To explore the possible therapeutic avenues in the setting of OGN overexpression in meningioma and its activation of mTOR and AKT, we tested the effect of a small molecule inhibitor of AKT (AKTVIII) on meningioma cells. Dose and time titration curves were established to determine the optimal drug dose for functional analysis (Fig. 6a). OGN-overexpressing cells demonstrated greater sensitivity to AKT inhibition, with diminished survival, compared to control cells at 48 h and 72 h (Fig. 6b). Protein pathway analysis by Western blot confirmed that AKT inhibition suppressed AKT activation as well as downstream mTOR phosphorylation at Ser2448 (Fig. 6c), the activation of mTOR substrates 4E-BP1, EIF4b, and eEF2K was also reduced (Additional file 2: Figure S2A). Interestingly, AKT inhibition also led to a reduction in OGN expression, suggestive of a reciprocal influence between OGN and AKT. In addition, NF2 siRNA significantly reduced NF2 protein expression and activated AKT while AKT inhibition conversely did not affect NF2 expression in OGN overexpressing cells which indicate AKT activation is the downstream signal of NF2 (Additional file 2: Figure S2). Addition of AKT inhibitor reduced the expression of the cell cycle marker cyclin B1, increased the expression of the autophagy marker LC3IIB, and suppressed phosphorylation of UNC-51-like kinase (ULK) at Ser757, a negative regulator in autophagy activation, supporting a biological cascade leading to the consequent cell death. The presence of autophagy in OGN meningioma cells treated with AKT inhibitor was confirmed by immunoreactivity for LC3IIB (Fig. 6d).

## Discussion

Large scale genomic and epigenomic profiling has provided new insights into meningioma oncogenesis in recent years [4–7]. However, much remains unknown about the specific signaling cascades leading to tumor growth. We investigated the effects of OGN, a critical regulator of bone and cardiovascular development, in meningioma proliferation, its association with other known signaling pathways involved in meningioma development, and explored the treatment options for OGN-expressing meningioma cells. Although previous work has identified OGN expression across a range of cancers, its oncogenic role has not been previously described [23, 24]. We found that OGN overexpression increased cell proliferation, that knockdown reduced cell growth, and that cells expressing high levels of OGN were sensitive to AKT inhibition; thereby, establishing for the first time, a potential mitogenic effect for OGN in meningioma.

Our results implicate OGN as an important mitogenic factor in meningioma growth across a range of tumor subtypes. Inhibitors of recently identified meningioma oncogenes, including *AKT1, SMO,* and *PIK3CA,* are now in clinical trial for recurrent and progressive meningiomas. However, alterations to these pathways occur in only a small subset of meningiomas, which are almost exclusively grade I [20]. We observed robust OGN expression across a wide range of meningioma histological subtypes and grades, at significantly higher levels than eight other CNS tumors and normal brain, posing OGN as an appealing target for those meningiomas which currently do not have pharmacologic treatment options.

OGN participates in meningioma formation in a distinct manner from previously identified genetic alterations. Genetic mutations and chromosomal aberrations identified in meningiomas implicate a critical role for cell cycle promotion, the Hedgehog pathway, and the PI3K/Akt pathway in tumorigenesis [4, 5]. We observed no mutation or copy number alterations in *OGN* in an extensive series of meningioma samples and cell lines that underwent next-generation sequencing [20, 22], which suggests that wildtype OGN may function as a co-activator of signals that promote meningioma growth and neoplasia formation.

Interestingly, we identified synergy between OGN and previously identified oncogenic signaling pathways in meningioma. OGN appears to downregulate NF2, the canonical tumor suppressor altered in approximately half of meningiomas. In addition, meningiomas with high OGN mRNA levels harbor more frequent *NF2* or chr22 loss than those with low OGN mRNA levels, and OGN protein expression was higher in meningiomas with *NF2* loss. Furthermore, AKT inhibitors had a selective effect on cells with high levels of OGN, even in the absence of oncogenic *AKT1* mutations, likely due to the fact that OGN expression increases p-AKT levels. Inhibition of AKT did not alter NF2 expression in OGN cells, while knockdown of NF2 significantly reduced AKT activation, suggesting that AKT activation could be the downstream signal of NF2 in OGN overexpressing cells. The sensitivity of OGN-expressing meningioma cells to AKT inhibition suggests that OGN expression may serve as an addition biomarker to stratify the response of aggressive meningiomas to AKT inhibitors.

## Conclusion

We identify OGN as a novel oncogene in meningioma proliferation. AKT inhibition reduces OGN protein levels in meningioma cells, with a concomitant increase in cell death. This study lays a foundation for the incorporation of OGN expression into personalized treatment for meningiomas.

## Additional files

Additional file 1: Figure S1. OGN mRNA expression in tonsil, as a negative control of OGN RNAscope. (PDF 1195 kb)

Additional file 2: Figure S2. AKT inhibitor reduces mTOR downstream signaling activation, but without effects on NF2. (**A**) Addition of AKT inhibitor (AKTi) to OGN overexpressing cells led to decreased activation of the mTOR downstream signals 4E-BP1, EIF4b, and eEF2K without altering NF2 expression. (**B**) Knockdown of NF2 with siRNA significantly reduced NF2 protein expression and increased AKT activation in OGN cells. *p < 0.05. (PDF 1796 kb)

## Abbreviations

4E-BP1: Eukaryotic translation initiation factor 4E-binding protein 1
AKT: v-akt murine thymoma viral oncogene homolog
BAP1: BRCA1 Associated Protein 1
COSMIC: Catalogue of somatic mutations in cancer
eEF2K: Eukaryotic Elongation Factor 2 Kinase
eIF4B: Eukaryotic translation initiation factor 4B
IHC: Immunohistochemistry
KLF4: Krupplelike factor 4
LC3IIB: Microtubule-associated protein light chain 3 IIB
mTORC1: Mammalian target of rapamycin complex 1
NF2: Neurofibromatosis
OGN: Osteoglycin
PIK3CA: Phosphoinositide-3-kinase catalytic alpha polypeptide
POLR2A: RNA polymerase II subunit A
qPCR: Quantitative real-time PCR
SMARCB1: SWI/SNF related, matrix associated, actin dependent regulator of chromatin, subfamily b, member 1
SMO: Smoothened
SUFU: Homolog of suppressor of fused
TERT: Telomerase reverse transcriptase
TRAF7: TNF receptor-associated factor 7
ULK: UNC-51-like kinase

## References

[1] Wiemels J, Wrensch M, Claus EB (2010). Epidemiology and etiology of meningioma. J Neuro-Oncol.

[2] Louis DN, Ohgaki H, Wiestler OD, Cavenee WK, Ellison DW, Figarella-Branger D, Perry A, Reifenberger G, von Deimling A. WHO classification of tumors of the central nervous system. 4th ed. France: International Agency for Research on Cancer Lyon; 2016. 10.1007/s00401-016-1545-1 27157931

[3] Rouleau GA, Merel P, Lutchman M, Sanson M, Zucman J, Marineau C, Hoang-Xuan K, Demczuk S, Desmaze C, Plougastel B (1993). Alteration in a new gene encoding a putative membrane-organizing protein causes neuro-fibromatosis type 2. Nature.

[4] Brastianos PK, Horowitz PM, Santagata S, Jones RT, McKenna A, Getz G, Ligon KL, Palescandolo E, Van Hummelen P, Ducar MD (2013). Genomic sequencing of meningiomas identifies oncogenic SMO and AKT1 mutations. Nat Genet.

[5] Abedalthagafi M, Bi WL, Aizer AA, Merrill PH, Brewster R, Agarwalla PK, Listewnik ML, Dias-Santagata D, Thorner AR, Van Hummelen P (2016). Oncogenic PI3K mutations are as common as AKT1 and SMO mutations in meningioma. Neuro-Oncology.

[6] Clark VE, Erson-Omay EZ, Serin A, Yin J, Cotney J, Ozduman K, Avsar T, Li J, Murray PB, Henegariu O (2013). Genomic analysis of non-NF2 meningiomas reveals mutations in TRAF7, KLF4, AKT1, and SMO. Science.

[7] Shankar GM, Abedalthagafi M, Vaubel RA, Merrill PH, Nayyar N, Gill CM, Brewster R, Bi WL, Agarwalla PK, Thorner AR, et al. Germline and somatic BAP1 mutations in high-grade rhabdoid meningiomas. Neuro-Oncology. 2017;19:535–45. 10.1093/neuonc/now235 PMC5464371 28170043

[8] Goutagny S, Nault JC, Mallet M, Henin D, Rossi JZ, Kalamarides M (2014). High incidence of activating TERT promoter mutations in meningiomas undergoing malignant progression. Brain Pathol.

[9] Clark VE, Harmanci AS, Bai H, Youngblood MW, Lee TI, Baranoski JF, Ercan-Sencicek AG, Abraham BJ, Weintraub AS, Hnisz D (2016). Recurrent somatic mutations in POLR2A define a distinct subset of meningiomas. Nat Genet.

[10] Harmanci AS, Youngblood MW, Clark VE, Coskun S, Henegariu O, Duran D, Erson-Omay EZ, Kaulen LD, Lee TI, Abraham BJ (2017). Integrated genomic analyses of de novo pathways underlying atypical meningiomas. Nat Commun.

[11] James MF, Han S, Polizzano C, Plotkin SR, Manning BD, Stemmer-Rachamimov AO, Gusella JF, Ramesh V (2009). NF2/merlin is a novel negative regulator of mTOR complex 1, and activation of mTORC1 is associated with meningioma and schwannoma growth. Mol Cell Biol.

[12] Tanaka K, Matsumoto E, Higashimaki Y, Katagiri T, Sugimoto T, Seino S, Kaji H (2012). Role of osteoglycin in the linkage between muscle and bone. J Biol Chem.

[13] Fernandez B, Kampmann A, Pipp F, Zimmermann R, Schaper W (2003). Osteoglycin expression and localization in rabbit tissues and atherosclerotic plaques. Mol Cell Biochem.

[14] Shanahan CM, Cary NR, Osbourn JK, Weissberg PL (1997). Identification of osteoglycin as a component of the vascular matrix. Differential expression by vascular smooth muscle cells during neointima formation and in atherosclerotic plaques. Arterioscler Thromb Vasc Biol.

[15] Petretto E, Sarwar R, Grieve I, Lu H, Kumaran MK, Muckett PJ, Mangion J, Schroen B, Benson M, Punjabi PP (2008). Integrated genomic approaches implicate osteoglycin (Ogn) in the regulation of left ventricular mass. Nat Genet.

[16] Jones RT, Abedalthagafi MS, Brahmandam M, Greenfield EA, Hoang MP, Louis DN, Hornick JL, Santagata S. Cross-reactivity of the BRAF VE1 antibody with epitopes in axonemal dyneins leads to staining of cilia. Modern pathology. 2015;28:596–06. 10.1038/modpathol.2014.150 25412847

[17] Ruifrok AC, Johnston DA (2001). Quantification of histochemical staining by color deconvolution. Anal Quant Cytol Histol.

[18] Lee WH (1990). Characterization of a newly established malignant meningioma cell line of the human brain: IOMM-Lee. Neurosurgery.

[19] Johanns TM, Fu Y, Kobayashi DK, Mei Y, Dunn IF, Mao DD, Kim AH, Dunn GP (2016). High incidence of TERT mutation in brain tumor cell lines. Brain Tumor Pathol.

[20] Bi WL, Greenwald NF, Abedalthagafi M, Wala J, Gibson WJ, Agarwalla PK, Horowitz P, Schumacher SE, Esaulova E, Mei Y, et al. Genomic landscape of high-grade meningiomas. NPJ Genom Med. 2017;2. doi:10.1038/s41525-017-0014-7. 10.1038/s41525-017-0014-7 PMC5506858 28713588

[21] Ruttledge MH, Sarrazin J, Rangaratnam S, Phelan CM, Twist E, Merel P, Delattre O, Thomas G, Nordenskjold M, Collins VP (1994). Evidence for the complete inactivation of the NF2 gene in the majority of sporadic meningiomas. Nat Genet.

[22] Mei Y, Bi WL, Greenwald NF, Agar NY, Beroukhim R, Dunn GP, Dunn IF (2017). Genomic profile of human meningioma cell lines. PLoS One.

[23] Orr B, Riddick AC, Stewart GD, Anderson RA, Franco OE, Hayward SW, Thomson AA (2012). Identification of stromally expressed molecules in the prostate by tag-profiling of cancer-associated fibroblasts, normal fibroblasts and fetal prostate. Oncogene.

[24] Zheng CX, Zhao SX, Wang P, Yu HM, Wang CF, Han B, Su B, Xiang Y, Li XS, Li SX (2009). Different expression of mimecan as a marker for differential diagnosis between NSCLC and SCLC. Oncol Rep.

