# Supplementary figures and images for: Osteoglycin promotes meningioma development through downregulation of NF2 and activation of mTOR signaling
Source: Cell Commun Signal. 2017 Sep 18;15:34. doi: 10.1186/s12964-017-0189-7 (PMC5604305; doi:10.1186/s12964-017-0189-7)

Negative control

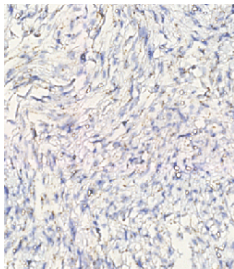

Tonsil

Supplement: Supplementary file 1 — OGN mRNA expression in tonsil, as a negative control of OGN RNAscope. (PDF 1195 kb) [file 12964_2017_189_MOESM1_ESM.pdf]

A

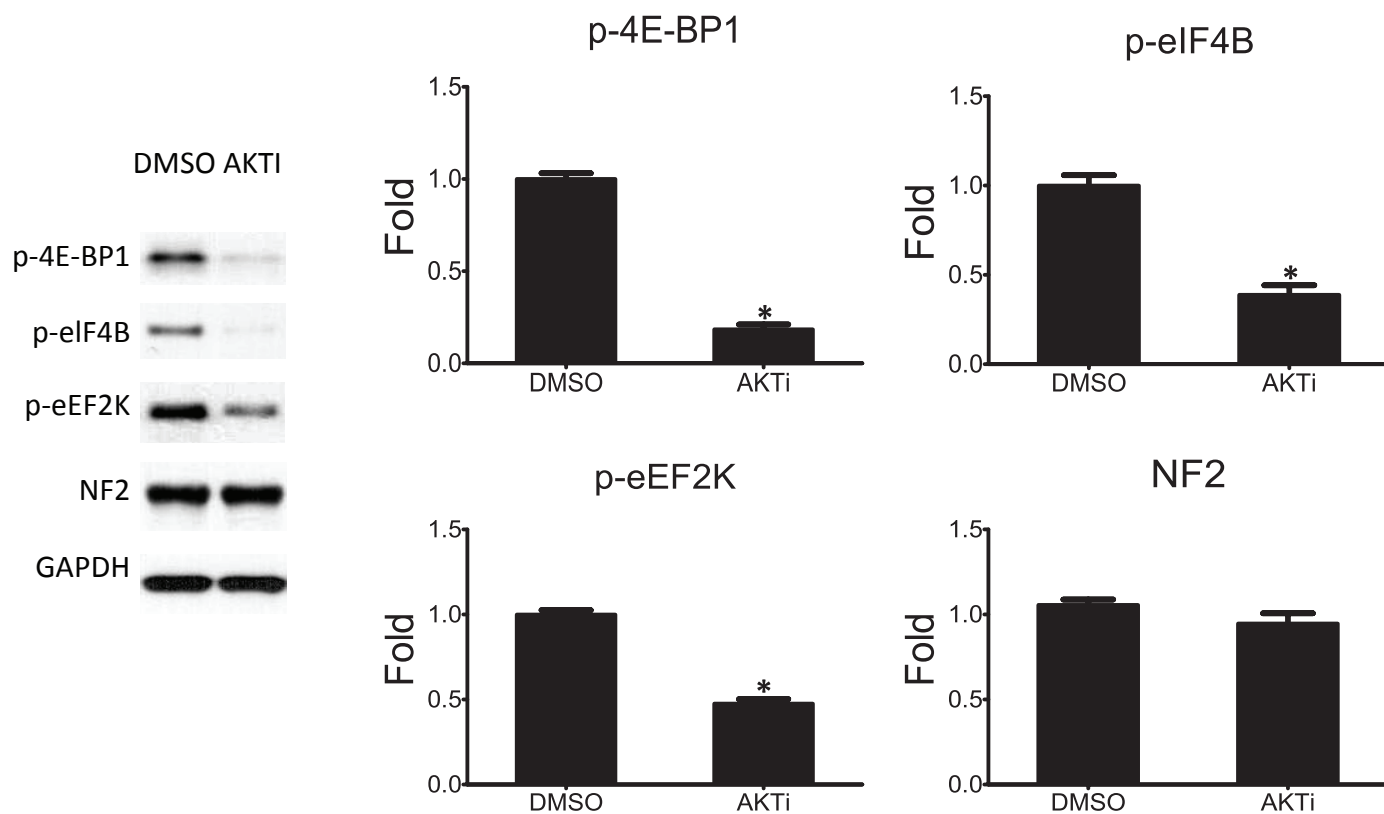

B

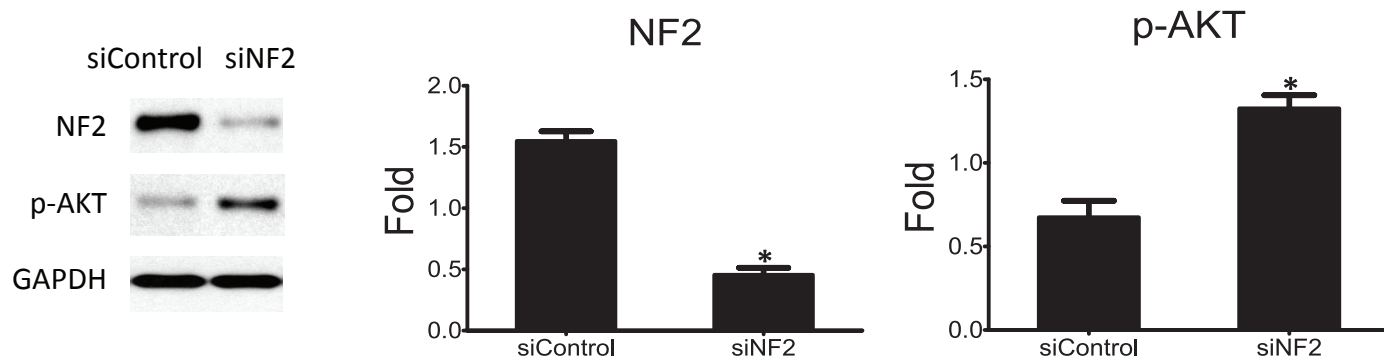

Supplement: Supplementary file 2 — AKT inhibitor reduces mTOR downstream signaling activation, but without effects on NF2. (A) Addition of AKT inhibitor (AKTi) to OGN overexpressing cells led to decreased activation of the mTOR downstream signals 4E-BP1, EIF4b, and eEF2K without altering NF2 expression. (B) Knockdown of NF2 with siRNA significantly reduced NF2 protein expression and increased AKT activation in OGN cells. *p < 0.05. (PDF 1796 kb) [file 12964_2017_189_MOESM2_ESM.pdf]
